# Audit of Lithium in a Psychiatry of Old Age Service

**DOI:** 10.1192/bjo.2022.426

**Published:** 2022-06-20

**Authors:** Salah Ateem

**Affiliations:** Cavan and Monaghan Mental Health Service, Monaghan, Ireland

## Abstract

**Aims:**

The aim of this audit is to compare our prescribing and monitoring practices in the Mental Health Service for the Elderly team (MHSE) in County Monaghan with current NICE guidelines (National Institute for Health and Care Excellence, UK). Lithium is used in the management of Bipolar Affective Disorder (BPAD) and refractory Depression in the elderly and across other patient groups. The elderly population is more vulnerable generally than other patient groups to adverse effects and toxicity from Lithium including at therapeutic doses. This is due to the increased likelihood of having other medical morbidities, interaction with other medications and the higher prevalence of renal impairment.

**Methods:**

The audit duration was from the beginning of April to the end of June 2021. Data were collected for demographic variables and for therapeutic variables such as Lithium dose, serum Lithium level, adverse effects due to Lithium, weight and signs of Lithium toxicity. Re-audit was completed during the month of June 2021.

**Results:**

Ten patients attending the MHSE team were prescribed Lithium at the time of the audit and were included in the audit. 60% were females and 40% were males. The mean age was 77.3 years. 50% had a Depressive disorder and 50% had a diagnosis of BPAD. The mean Lithium dose was 310 mg and the mean serum Lithium level was 0.5mmol/L. All 3-monthly Lithium levels were completed. 100% were provided with information booklets, record books and BMI recorded. 60% of six-monthly Lithium levels were completed. 20% of six-monthly bloods were completed but not documented. There were eight patients prescribed Lithium in the re-audit. 62% were females and 38% were males. The mean age was 78.8 years. 75% were diagnosed with refractory Depression and 25% with BPAD. The mean Lithium dose was 337.5 mg and the mean serum Lithium level was 0.6mmol/L. 100% of patients completed their three and six-monthly serum Lithium levels and documentation was complete for 100% of patients.

**Conclusion:**

We recommend the establishment of a Lithium Clinic to ensure proper monitoring of this group. This includes clear pathways for patients to have their bloods taken (GP or hospital), pro forma reminder letters for GPs and patients, a recording table for blood results and physical variables in the patient file, alert cards and the provision of written information about Lithium for patients and carers.

